# Money Matters: Anticipated Expense of In-Person Obstetrics and Gynecology Fellowship Interviews Has Greater Impact for Underrepresented in Medicine and Women Applicants

**DOI:** 10.1089/whr.2021.0114

**Published:** 2022-08-04

**Authors:** Christine A. Heisler, Sylvia Botros-Brey, Hanzhang Wang, Ann Tran, Bertille Gaigbe-Togbe, Ava Leegant, Anne Hardart

**Affiliations:** ^1^ Division of Female Pelvic Medicine and Reconstructive Surgery, Department of Obstetrics and Gynecology, University of Wisconsin School of Medicine and Public Health, Madison, Wisconsin, USA.; ^2^ Division of Female Pelvic Medicine and Reconstructive Surgery, Department of Urology, University of Wisconsin School of Medicine and Public Health, Madison, Wisconsin, USA.; ^3^ Division of Female Pelvic Medicine and Reconstructive Surgery, Department of Urology, University of Texas Health San Antonio, Joe R and Theresa Lozano Long School of Medicine, San Antonio, Texas, USA.; ^4^ Division of Female Pelvic Medicine and Reconstructive Surgery, Department of Medical Education, University of Texas Health San Antonio, Joe R and Theresa Lozano Long School of Medicine, San Antonio, Texas, USA.; ^5^ Division of Female Pelvic Medicine and Reconstructive Surgery, Department of Obstetrics, Gynecology, and Reproductive Science, Mount Sinai Health System, Icahn School of Medicine, New York, New York, USA.; ^6^ Division of Female Pelvic Medicine and Reconstructive Surgery, Department of Obstetrics and Gynecology, Albert Einstein College of Medicine, Montefiore Medical Center, Bronx, New York, USA.

**Keywords:** obstetrics and gynecology, fellowship, financial implications, gender equity, racial equity

## Abstract

**Background:**

Much of the expense of pursuing subspecialty training in obstetrics and gynecology (ObGyn) is due to in-person fellowship interviews. Although interviews were converted to a virtual platform for the 2020 fellowship interview season in response to the COVID-19 pandemic, candidates anticipated in-person interview expenses at the time of their application. It is unknown whether financial considerations influenced candidates' decision to pursue fellowship training. This study aimed to evaluate the financial impact of anticipated in-person fellowship interviews among applicants of ObGyn subspecialties.

**Materials and Methods:**

This was a planned secondary analysis of a survey administered during the 2020 interview season to evaluate the effectiveness of virtual ObGyn fellowship subspecialty interviews in creating a rank list. Information was obtained about anticipated and actual interview costs, the need for securing additional funding and whether financial considerations influenced the decision to apply for fellowship.

**Results:**

In total, 158 participants enrolled in the 2020 National Resident Matching Program for ObGyn fellowship programs (48%) completed the web-based survey. Women and Black fellowship applicants were more likely than men (*p* = 0.044) and White applicants (*p* = 0.014) to endorse a need to secure additional funding for in-person fellowship interviews. In addition, Hispanic and Black applicants were more likely than White applicants to report that the financial impact of fellowship interviews influenced the decision to apply “somewhat” or “to a great extent” (*p* = 0.025 and *p* < 0.001, respectively).

**Conclusions:**

The costs of applying to ObGyn fellowship programs may disproportionately affect women and underrepresented in medicine applicants. By reducing a financial barrier, virtual interviews may help promote greater gender and racial and ethnic diversity in ObGyn subspecialty pursuit.

## Introduction

Diversity, equity, and inclusion in medicine are critical factors in the delivery of high-quality health care.^1,2^ Diversity in health care improves access and patient care outcomes, strengthens teams, and drives innovation.^3–6^ Representation of women and individuals from ethnic and racial groups that are underrepresented in medicine (URiM) declines from obstetrics and gynecology (ObGyn) residency to subspecialty fellowship programs.^7^

Fellowship training is pursued by >40% of ObGyn residents,^8^ with the average expenditure of the interview process estimated at $6000 per candidate.^9^ These expenses are similar to in-person interview costs reported for other specialties.^10,11^ Alarmingly, greater candidate expenditure during the interview process was the only significant predictor of matching in a fellowship program, despite honors, elective rotations, or outstanding references.^12^

Travel restrictions for the 2020 interview season due to the COVID-19 pandemic required conversion of planned in-person fellowship interviews to a virtual interview platform. Previous single-specialty studies have associated virtual interviews with lower applicant costs.^13^ However, the financial implications of the rapid and wide-spread adoption of virtual interviews on candidates compared with planned in-person interviews are unknown.

The objective of this study was to explore specific financial considerations that may influence applicants' pursuit of ObGyn subspecialty fellowships and the impact of virtual interviews on expenses during the COVID-19 pandemic.

## Materials and Methods

This was a planned secondary analysis of an IRB-exempt cross-sectional study of ObGyn fellowship applicants and their experiences with virtual interviewing (A. Tran et al., under review). The parent study methods have previously been described.^14^ The survey was created and edited by the investigators' team according to the Checklist for Reporting Results of Internet ESurveys (CHERRIES) criteria.^15^

The survey was distributed during the 2020 interview season to a convenience sample of 330 ObGyn fellowship applicants from complex family planning, female pelvic medicine and reconstructive surgery, gynecologic oncology, maternal–fetal medicine, and minimally invasive gynecologic surgery. Candidates were invited to participate through e-mail, and study data were collected through REDCap.^16^

Participation was voluntary and anonymous, with consent obtained before initiation of the survey. Demographic data were collected. In addition, the survey included specific financial questions regarding estimated and actual expenses, need for incurring additional debt (personal loans or credit cards) as a result of fellowship pursuit, and the degree to which financial considerations influenced the decision to apply for fellowship.

Two sample *t*-test and Wilcoxon rank-sum test were used for comparing continuous variables; categorical variables were compared using chi-square and one way analysis of variance test. Data were analyzed using Stata statistical software (Release 15., College Station, TX). Statistical significance was defined as *p* < 0.05. Statistical analysis was completed in January 2021.

## Results

A total of 330 emailed surveys were received by applicants, and 158 provided answers to questions pertaining to financial impact (48%). Demographic, geographic, training environment, fellowship, and financial impact variables are presented in Table 1. The majority of respondents were women [122/149 (82%)], younger than 30 years [52/149 (35%)], and non-Hispanic White [82/149 (55%)] from academic ObGyn residency programs [110/149 (74%)]. A total of 27 respondents (of 158, 17%) identified as URiM, of which 18 were Hispanic and 9 were Black.

**Table 1.** Demographic Characteristics for Respondents by Fellowship Subspecialty, *N* (%)

	Complex family planning *N* = 18	Female pelvic medicine and reconstructive surgery *N* = 42	Gynecologic oncology *N* = 18	Maternal–fetal medicine *N* = 51	Minimally invasive gynecologic surgery *N* = 29
Age, years					
<30	11 (64.7)	23 (57.5)	11 (61.1)	24 (51.1)	17 (63.0)
30–34	1 (5.9)	1 (2.5)	0 (0)	4 (8.5)	3 (11.1)
35–39	5 (29.4)	15 (37.5)	6 (33.3)	19 (40.4)	7 (25.9)
Gender					
Female	16 (94.1)	31 (77.5)	15 (83.3)	37 (78.7)	23 (85.2)
Male	1 (5.9)	8 (20.0)	2 (11.1)	10 (21.3)	4 (14.8)
Prefer not to answer	0 (0)	1 (2.5)	1 (5.6)	0 (0)	0 (0)
Race					
Asian	3 (23.1)	8 (21.1)	4 (22.2)	9 (20.9)	9 (36.0)
Black	1 (7.7)	1 (2.6)	0 (0)	3 (7.0)	3 (12.0)
Mixed/some other category	0 (0)	1 (2.6)	2 (11.1)	2 (4.7)	1 (4.0)
White	9 (69.2)	24 (63.2)	11 (61.1)	29 (67.4)	12 (48.0)
Prefer not to answer	0 (0)	4 (10.5)	1 (5.6)	0 (0)	0 (0)
Ethnicity					
Hispanic	4 (22.2)	2 (4.8)	0 (0)	8 (15.7)	4 (13.8)
Non-Hispanic	14 (77.8)	40 (95.2)	18 (100)	43 (84.3)	25 (86.2)
Location					
Midwest	6 (35.3)	N/A	6 (35.3)	11 (23.4)	6 (23.1)
Northeast	4 (23.5)	N/A	6 (35.3)	20 (42.6)	8 (30.8)
South	3 (17.7)	N/A	4 (23.5)	13 (27.7)	7 (26.9)
West	4 (23.5)	N/A	0 (0)	3 (6.4)	3 (11.5)
Prefer not to answer	0 (0)	N/A	1 (5.9)	0 (0)	2 (7.7)
Training program					
ObGyn—academic	15 (88.2)	29 (72.5)	15 (83.3)	33 (70.2)	18 (66.7)
ObGyn—community	1 (5.9)	6 (15.0)	2 (11.1)	12 (25.5)	5 (18.5)
ObGyn—other	1 (5.9)	0 (0)	1 (5.6)	0 (0)	3 (11.1)
Urology—academic	0 (0)	5 (12.5)	0 (0)	0 (0)	0 (0)
Prefer not to answer	0 (0)	0 (0)	0 (0)	2 (4.3)	1 (3.7)

N/A, question was not asked in surveys for female pelvic medicine and reconstructive surgery applicants.

ObGyn, obstetrics and gynecology.

Although the numbers are small, over half of the Black applicants [5/9 (56%); *p* = 0.014] and almost a quarter of women [28/122 (23%); *p* = 0.044] endorsed a need to fund interviews through personal loans or credit cards (see Table 2). Only two men [of 25, or 8%] responded with a similar need for funding interviews. A total of 18 respondents [of 149, 12%] stated finances influenced their decision to apply to fellowship “somewhat” or “to a great extent” (see Table 3). Of those endorsing a financial impact to pursue fellowship, Hispanic [4/18 (22%); *p* = 0.025] and Black [3/9 (33%); *p* < 0.001] applicants were significantly more affected. URiM candidates were nearly three times more likely than non-URiM candidates to state that finances affected the decision to apply to fellowship [7/27 (25%) vs. 11/122 (9%)].

**Table 2.** Survey Question: Did You Plan on Taking Loans or Getting a New Credit Card to Finance Your Interviews Before Conversion to Virtual Format?

	No	Yes	Not sure	Total	*p*	*p* (no vs. yes)
Race, ethnicity						
Hispanic	14 (77.78%)	3 (16.67%)	1 (5.56%)	18 (100%)	0.806	0.611
Black	4 (44.44%)	5 (55.56%)	0 (0%)	9 (100%)	0.025	0.014
Native American	0 (0%)	0 (0%)	1 (100%)	1 (100%)	0.003	N/A
Asian	29 (85.29%)	1 (2.94%)	4 (11.76%)	34 (100%)	0.013	0.005
White	60 (68.97%)	20 (22.99%)	7 (8.05%)	87 (100%)	0.703	0.402
Others	3 (60%)	2 (40%)	0 (0%)	5 (100%)	0.486	0.339
Prefer not to answer	3 (60%)	1 (20%)	1 (20%)	5 (100%)	0.599	0.902
Gender						
Female	85 (69.67%)	28 (22.95%)	9 (7.38%)	122 (100%)	0.049	0.044
Male	21 (84%)	2 (8%)	2 (8%)	25 (100%)		
Prefer not to answer	0 (0%)	1 (50%)	1 (50%)	2 (100%)		

**Table 3.** Survey Question: Please Indicate to What Extent the Anticipated Financial Impact of Interviews Influenced Your Decision to Apply (Categorized by Race/Ethnicity)

	Not at all	Very little	Somewhat	To a great extent	Total	*p*
Hispanic	13 (72.22%)	1 (5.56%)	2 (11.11%)	2 (11.11%)	18 (100%)	0.025
Black	3 (33.33%)	3 (33.33%)	1 (11.11%)	2 (22.22%)	9 (100%)	<0.001
Native American	1 (100%)	0 (0%)	0 (0%)	0 (0%)	1 (100%)	0.954
Asian	29 (85.29%)	3 (8.82%)	2 (5.88%)	0 (0%)	34 (100%)	0.437
White	67 (77.01%)	10 (11.49%)	10 (11.49%)	0 (0%)	87 (100%)	0.179
Others	4 (80%)	0 (0%)	1 (20%)	0 (0%)	5 (100%)	0.731
Prefer not to answer	3 (60%)	2 (40%)	0 (0%)	0 (0%)	5 (100%)	0.280

Approximately half of the respondents (73/150) estimated in-person interview expenses to exceed $6000. However, the median amount spent on the entire virtual interview process was $200. Nearly all applicants (146/148, 99%) cited reduced costs as an advantage of virtual interviews.

## Discussion

These results suggest that women and Black applicants may be more likely than men or White applicants to incur additional debt to attend in-person fellowship interviews, and that financial considerations are more likely to influence Hispanic and Black fellowship applicants than their White colleagues.

The financial burden of in-person interviews has been well documented across multiple specialties.^9,10,12^ In fact, the mean expense per in-person surgical subspecialty fellowship interview was $458–600^11,17,18^ with some candidates reporting total cost to pursue fellowship exceeding $20,000.^19^ This is in stark contrast to the total expense of $200 for all fellowship virtual interviews that was identified in the original study. These data confirm the cost savings of virtual interviews and that applicants see this as a significant advantage.

The effect of virtual interviews on diversity has not previously been investigated. Nwora et al.^20^ suggested virtual interviews could have disproportionately negative effects on URiM students and residents due to implicit bias. Specific sources of bias may include technology challenges and visual cues of the applicants' personal living situation.^21,22^ Despite these hypotheses, the actual impact of virtual interviews on diversity has not been studied.

This study quantified the financial considerations and repercussions of anticipated in-person fellowship interviews for women and URiM applicants. The lack of financial resources is a recognized deterrent to career development for women and URiM trainees.^23^ Widespread adoption of a virtual interview platform is a feasible way to remove one barrier to advancing diversity.

Since respondents applied to fellowship before the confirmation of a virtual interview platform, they anticipated the expense of interviewing in person. This study did not survey residents for whom the financial impact of applying to fellowship may have been a deterrent. Gauging interest in fellowship pursuit for junior ObGyn residents and understanding the factors that influence this decision are critical to ensuring inclusivity in the subspecialties.

This study has specific limitations. Residents were not surveyed, so this study may actually underestimate the financial burden for women and URiM fellowship applicants, as those who may be more seriously impacted by finances might choose not to pursue fellowship. Although five ObGyn subspecialties were represented in the survey, it was not administered to pediatric and adolescent gynecology or reproductive endocrinology and infertility fellowship candidates, so results may not be applicable to these subspecialties.

Access to the survey link was closed before the match to limit confounding results in the primary study that shortened the time available to complete the survey and may have contributed to the lower number of respondents. However, the number of URiM respondents was 27 [of 158 (17%)], which is reflective of the number of URiM residents in ObGyn [864/5486 (16%) in 2020].^24^

Despite these limitations, this study has specific strengths. This multicenter collaborative study included a wide range of ObGyn subspecialties with regional variation. Importantly, the survey utilized in this study was rigorously constructed and internally validated before implementation and the financial information was novel. The gender and racial/ethnic demographics of the survey respondents were reflective of the general composition of ObGyn trainees.

## Conclusions

Women and URiM applicants are disproportionately impacted by financial considerations when applying to fellowship programs. The associated lower expenses of the virtual interview platform may improve gender and racial equity for ObGyn subspecialties.

## Abbreviations Used

ObGyn: obstetrics and gynecology
URiM: underrepresented in medicine
